# Evaluation of Self-Concept in the Project for People with Intellectual Disabilities: “We Are All Campus”

**DOI:** 10.3390/jintelligence9040050

**Published:** 2021-10-20

**Authors:** María Luisa Belmonte Almagro, Abraham Bernárdez-Gómez

**Affiliations:** 1 Department of Theory and History of Education, Universidad de Murcia, 30120 Murcia, Spain; marialuisa.belmonte@um.es; 2 Department of Didactics and School Organisation, Universidad de Murcia, 30120 Murcia, Spain

**Keywords:** self-concept, intellectual disability, university, inclusion, SWOT

## Abstract

The inclusion of people with disabilities, intellectual in the case that concerns this research, has been one of the main concerns of society in recent years. The University of Murcia has launched the “We are all Campus” program in order to facilitate the inclusion of this group from a training perspective. Being aware of the influence of self-concept in such inclusion, this research aims to analyze the influence of the self-concept of people with intellectual disabilities in their expectations of inclusion. For this purpose, 18 subjects were asked to carry out a SWOT analysis, assessing the situation in which they find themselves through a qualitative perspective and a phenomenological design. The research reveals, among other conclusions, how important personal development is to them, especially by generating autonomy in their daily routines, and also the relevance of their relationships to feel socially included.

## 1. Introduction

Inclusive education was originally intended for young students (Desombre et al. 2019). Therefore, the implementation of the principles of inclusive education in higher education implies a challenge (Moriña 2017). Since the creation of the convention relies on the Rights of Persons with Disabilities, there has been a change in perspective towards a social model, which advocates the understanding of disability as a social construction and not as a personal characteristic (Manzanera and Brändle 2019). For decades, disability struggled to find a place in the world of educational research, given the need to build new social pillars (Rocco 2011). The primary objective was to redefine misconceptions about disability hierarchies, as well as the relationship between people with and without disabilities, as descriptive terms (Vehmas and Watson 2014).

As more students with disabilities successfully complete their early education, the need to move towards inclusive practices within higher education is increasing (Moriña 2017). Historically, university education and the group of students with intellectual disabilities have not been compatible entities (Brewer and Movahedazarhouligh 2019). In recent years, higher education institutions, on an upward scale, have offered opportunities for students with intellectual disabilities to participate in campus life (Ryan et al. 2019). This has meant a significant advance in terms of educational inclusion (Moriña et al. 2020a), which has been reflected in the increase in studies on the response of higher education to this new situation of inclusion of young people with disabilities in the University (Claiborne et al. 2010; Hopkins 2011; Moriña 2017; Moriña and Perera 2018; Mullins and Preyde 2013).

Independence and autonomy are key goals for people with intellectual disabilities (Roldán-Álvarez et al. 2021). The importance of related work focused on making higher education more accessible for students with disabilities (Putnam et al. 2016), but there is little knowledge about the prevalence of higher education teaching about accessibility or faculty’s perceived barriers to teaching accessibility (Shinohara et al. 2018).

Inclusion is based on the normalization of the life of the student with special educational needs; however, once secondary education is completed, it is not usual to expect that people with a disability will continue their journey through the paths of higher education (Gillan and Coughlan 2010; Grigal and Hart 2010; Mock and Love 2012). Therefore, there is still a long way to go. On the other hand, employment is a crucial element to promote the development of the quality of life of people with intellectual disabilities (Ryan et al. 2019), despite the different challenges that this aspiration entails (Sandoval et al. 2018). Inclusion of the students into the labor market is one of the main keys to normalization (Manzanera and Ortiz 2017), along with their inclusion into society (Manzanera and Brändle 2019). In general, the situation of this particular group in respect of the labor market is worse than that of people without disabilities (Manzanera and Ortiz 2017; Ortiz and Olaz 2018).

Closely linked to the inclusion of the person with intellectual disabilities is the self-concept that this group expresses and how their passage through the university can lead to an improvement in it. It should be remembered that self-concept, as a system of cognitive structures to interpret and respond to the environment (Brewer and Movahedazarhouligh 2019; Manzanera and Brändle 2019) based on their attitudes, has four fundamental aspects:

- The self-concept of an affective nature, which is understood through the perception that a person manifests regarding their emotional development (Claiborne et al. 2010);
- The self-concept of an ethical nature, which is related to the honesty and vision of that person integrated into society with the civic values marked by it (Mullins and Preyde 2013);
- The self-concept of an autonomous nature, where the individual experience is perceived as the possibility of acting according to one’s own criteria (Fonseca et al. 2019);
- The self-concept related to self-realization, which reflects the achievement of planned goals and the state of the person in respect of her objectives (Mock and Love 2012).

Thus, in the inclusion of people with intellectual disabilities, the different experiences that these subjects face and the perspective from which students perceive different situations and experiences throughout their lives, as well as the expectations that they develop play a fundamental role over time. Factors related to own beliefs, self-understanding, and context (such as responsibilities, mentality, and personal experiences) play a very important role (Van-Mieghem et al. 2018), as well as procedural factors (such as the educational environment or leadership (Arnaiz et al. 2020), the perspective of oneself (Miñano et al. 2012; Dedrick et al. 2015), their motivation (Langørgen and Magnus 2018; Muro et al. 2018), and self-determination (Langørgen and Magnus 2018)).

In order to facilitate such inclusion, different institutions have developed training initiatives that meet this objective, in the case of the University of Murcia, the “We are all Campus” program (Belmonte et al. 2020).

### “We Are All Campus” Program

Within the framework of the Europe 2020 Strategy, designed to promote favorable conditions for a more competitive, job-generating, sustainable, and inclusive economy, it seems necessary to propose actions that reinforce the employment of the most disadvantaged sectors of the population.

The University of Murcia has set up a university degree called “We are all Campus”. This initiative wants to join the social effort to favor and facilitate the integration of young people with intellectual disabilities in the labor market. To this end, a training project for employment begins to be forged, trying to respond to the individual needs of this group by building comprehensive and personalized training itineraries so that young people with intellectual disabilities can participate as subjects of law in their community, fostering their capacities and job opportunities. They are integrated through the construction of comprehensive, accessible, and personalized training itineraries. 

The content approach is carried out in an applied and cross-sectional way, which includes a period of practices allowing students to interact with collaborating companies. The working methodology is based on collaborative, fully accessible, and flexible work groups and peer support with fixed goals, which are adapted to content requirements and being able to establish synergies between participants, chain activities, large group work, among others. 

“We are all Campus” means training to improve autonomy and socio-labor inclusion, bearing in mind that it develops motivation for learning and responsible task performance in order to say that the University of Murcia aims to establish a system for the inclusive training and community standardization.

The ambitious objectives of this program are to:

- Provide training aimed at improving autonomy and job preparation;
- Facilitate resources to increase labor and social inclusion of the group;
- Provide participants with comprehensive, humanistic, and multipurpose labor training, maximizing their access and maintenance possibilities in the job market;
- Establish an inclusive training and standardization system within the framework of the university community;
- Develop the motivation and interest of the participants in learning and performing the different tasks, with efficiency and responsibility.

Degree candidates are 18 young people, aged between 18 and 30, who meet the following requirements: enrolled in the Youth Guarantee System, have an intellectual disability equal to or greater than 33% officially recognized by the Murcian Institute of Social Action, possess autonomous movement capacity, and have basic academic skills with minimal literacy and numeracy skills.

In summary, some university sectors tend to alienate students with disabilities due to a lack of knowledge, training, and awareness (Bunbury 2018). Different studies confirm that this type of program will lead to a higher rate of employment and independent living for students with disabilities in the future (Ryan et al. 2019). However, it is necessary for universities to embrace inclusion, given that the person with a disability can be valuable, within a more mature university environment, which recognizes that all students are important and capable of learning, given appropriate attitudes and conditions (Moriña et al. 2020b).

Regardless of the attitudinal and structural barriers and obstacles that they may encounter on multiple occasions, people with disabilities begin to be welcomed in higher education institutions, becoming part of the university community (Björnsdóttir 2017), as it is already happening at the University of Murcia (Belmonte et al. 2020). For these reasons, higher education is a valid scenario where this student sector begins to be socially included, even allowing it to reinvent an identity that deteriorated during previous stages in its educational journey (Moriña 2017).

## 2. Objectives and Methodological Issues

### 2.1. Objectives

To carry out this research, we define the following general (GO) and specific objectives (SO):

- GO1. Analyze the influence of the “We are all Campus” program on the self-concept of people with intellectual disabilities in their inclusion expectations.
- SO1. Explore the perception of the factors that affect the self-concept of people with intellectual disabilities.
- SO2. Establish relationships with the categories that emerged between the different factors that affect the self-concept of people with disabilities.

### 2.2. Method

A qualitative methodological approach (Miles and Huberman 1984; Stoecker 1991) was adopted in this study, with a phenomenological research design focused on the description of life experience, which gives participants a voice so that they can be heard through their own experiences, openly communicated (Taylor et al. 2015). It is a way of approaching the work that leads the researcher to understand reality in a holistic way, observing the object studied from different perspectives (Stoecker 1991). It is a methodology where, in addition to the selected subjects, the researcher himself plays an important role, since one of the objectives that the researcher must achieve is to immerse oneself in the reality under study (Miles and Huberman 1984) and make it manifest. Thus, the role of the person conducting the research tries to understand and interpret the perceptions and meanings expressed by the subjects.

This research follows the design of case studies, since it is a way of responding to the need for a research strategy aimed at understanding the dynamics present in unique contexts (Taylor et al. 2015). The realization of this case study is an activity developed within the framework of the training program, so it is approved by the university, and its approval by the ethics commission is not necessary.

### 2.3. Context and Sample

The sample for the research developed in these lines was extracted from the University of Murcia which offers a degree that aims to facilitate entry into the labor market to people with intellectual disability. Eighteen young people, aged between 18 and 30, were enrolled in the 2020/2021 academic year. However, the sample may be small for a study. Hernández-Samfieri (2014) points out that, for research of these characteristics, case studies with 12 participants would be sufficient. In this case, 50% more are used than necessary. All of them met the following requirements: enrolled in the Youth Guarantee System, had an intellectual disability equal to or greater than 33% officially recognized by the Murcian Institute of Social Action, possessed autonomous movement capacity, and had basic academic skills with minimal literacy and numeracy skills.

### 2.4. Data Collection Tool, SWOT Analysis

In order to gather the necessary knowledge about the experience of users, considering their intellectual disability, the SWOT tool was used (Onion and Aranguren 2021; Teoli et al. 2019), which provides a situational analysis of the subjects where they analyze their characteristics internally. The technique used in the development of data collection is called projective. This has shown usefulness in situations where the person is explored from a dynamic point of view, since it is carried out from the inside of the individual and involves manifesting subjective elements of this, such as emotions, feelings, or ways of approaching situations (Miles and Huberman 1984). In this way, the SWOT was used to question them about the weaknesses, threats, strengths, and opportunities that they find due to their belonging to the group studied. Specifically, the subjects explained the meaning of each of the elements of the SWOT so that they could then report the weaknesses, threats, strengths, and opportunities provided by the university education they are undertaking in relation to their disability. Although the subjects presented an added difficulty in answering the question posed due to their deficiency, before beginning each of the points of the SWOT was explained in a simplified way so that no difficulty arose.

### 2.5. Data Analysis

It is known that there are multiple strategies to implement qualitative data analysis that were described over the last few years, and this is corroborated by different authors mentioned in the previous paragraphs. In this research, the analysis procedure established by Barton and Lazarsfeld (1961) was chosen because it meets the needs of the research developed, both for its depth and the tool used for the analysis, ATLAS.ti version 9. This method of analysis is developed in five levels from less to greater depth, through which the different meanings found in the data are extracted (Taylor et al. 2015). Those applied in this research are:

1. Analysis of observations that present the purpose of stimulating research. This was carried out at the beginning of it when the need was detected. Exposing the initial results of the encoding in absolute numbers.
2. Construction or use of descriptive systems. This consists of the coding of the extracted data to establish the categories. For this purpose, names of the SWOT matrix were deductively used as codes and, inductively, codes were created according to the topic addressed by the subjects. A more in-depth explanation of this is given in the descriptive analysis described in the next section.
3. Qualitative data suggesting relationships among variables were performed via an analysis of the co-occurrences between the emerged codes. These relationships are considered qualitative suggestions (quasi-statistics).
4. Matrix formulations expressed through semantic networks where specific interrelations are expressed and from which different patterns can be extracted in the subjects.
5. Qualitative analysis to support the theory focused on proposing trends and theoretically contrasting the different statements extracted from the analysis process.

## 3. Results and Discussion

**Figure 1:**
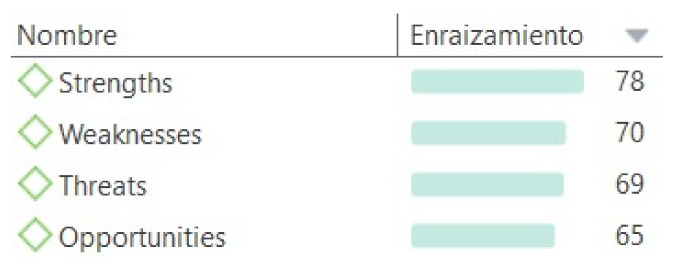
Rooting of the codes referred to the SWOT analysis^1^.

As a first result of the data analysis process, it is necessary to observe, in quantitative terms, the number of quotes that emerged in each of the pre-established codes and the different codes that emerged after the inductive categorization. Figure 1 reveals that the pre-established codes, which followed the categories in the SWOT analysis, show a similar response range with an anecdotal response variation between the one most indicated by the students, their strengths, and, the one with the lowest result, opportunities.

**Figure 2:**
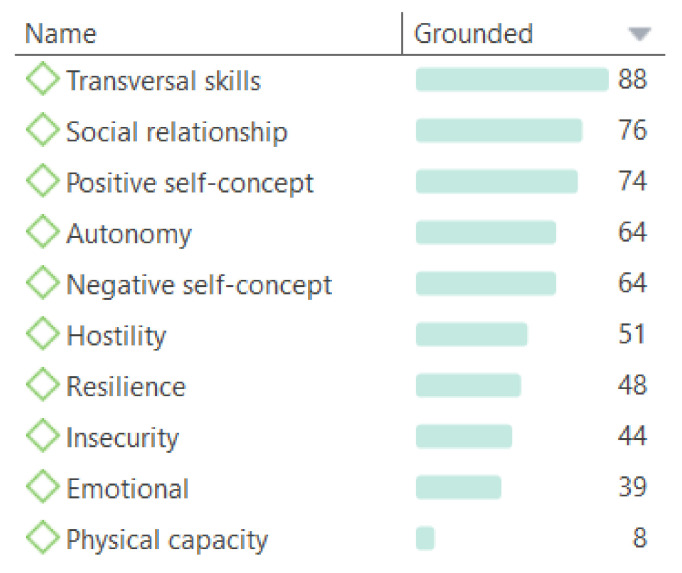
Rooting of the codes emerged inductively.

In the second part of the analysis, and as a result of the inductive coding, a total of ten categories emerged (Figure 2). Disparity is found in terms of their coding due to questions of analysis of various topics and aspects of the subjects’ lives. This highlights those different issues that (at least in the discourse) are most relevant to them, which were the following.

- Transversal skills: the largest of the categories in terms of quotes refers to different skills that are necessary for the daily life of the subjects, skills they express they still need, or skills they have already acquired, e.g., “I’m not good at handling money” (1:55)^2^.
- Social relationship: linked to quotes that refer to issues related to socialization with peers or other people, e.g., “I’m going to make friends at university” (1:28). This is highly valued by the whole sample.
- Positive/negative self-concept: the perception that subjects establish about themselves and their abilities, both negatively and positively, e.g., “I’m negative, I think everything will go wrong” (1:58).
- Autonomy: possible issues with dependency on other people or their freedom to act independently in society, e.g., “I usually do things for myself, I am independent” (1:31).
- Hostility: broad code used to indicate quotes in which harmful or aggressive aspects towards them are stated, such as bullying or lack of consideration by third parties, e.g., “Sometimes they laugh at me” (1:198).
- Resilience: adaptation with changes that provide positive results in adverse situations, in their case the cognitive deficiency itself, e.g., “I have succeeded in passing an exam” (1:5).
- Insecurity: any mention of situations of nervousness, fear, or incapacity in the face of a scenario or any problem caused by one’s perception of vulnerability, e.g., “I’m not sure if I will achieve my goals” (1:164).
- Emotional: code referring to expressions and quotes where subjects express ideas related to emotions and their stability in this sense. It was stated both positively and negatively, e.g., “Sometimes I feel excluded” (1:215).
- Physical capacity: used when referring to issues of this nature, e.g., “I have managed to walk alone, without help. I couldn’t do that before” (1:16).

**Figure 3:**
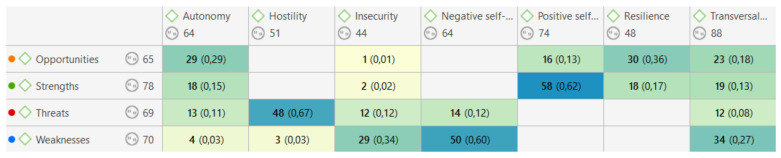
Co-occurrence coefficients between SWOT dimensions and emerged categories.

On the other hand, a broader analysis can be performed, in order to search for meanings of greater significance than the mere presence at the absolute level of the codes in the citations. Thus, when studying the different relationships that can be established between each of the codes, it is possible to observe different relationships that are established at the semantic level. Figure 3 shows the different coefficients of co-occurrence^3^ between each of the codes belonging to the SWOT dimensions and the rest of the coded categories. Some of them are shown and present a considerably high coefficient, establishing, at least initially, a possible relationship between these two codes.

In the first place, it can be highlighted how the self-concept has a strong link with, on the one hand, the weaknesses, in the case of the negative self-concept, showing a coefficient of co-occurrence (hereinafter @) of codes of @ 0.60, whereby, once investigated in the quotes that these categories have in common, it appears that the perceived weaknesses are related to the negative self-concept (“I’m not good at handling money” (1:55)). On the other hand, positive self-concept is closely linked to the perception of the strengths that the subjects have (@ 0.62) (“I have learned to do things by myself, which I did not know before” (1:4)).

This shows how the subjects studied in the present work value, a priori, the self-concept linked to their autonomy (Fonseca et al. 2019), where the different skills, exposed through their strengths and weaknesses, allow, or not, the capability of developing an independent life (Moriña and Perera 2018). This has shown that, when an environment that favors this independence is provided, subjects with intellectual disabilities present a better quality of life (Hopkins 2011), actively participating in their decisions and achieving greater development in their adult life (Arnaiz et al. 2019; Arnaiz et al. 2020; Claiborne et al. 2010).

Following this direction, other categories showed a consistent relationship with the different dimensions of the SWOT. The main opportunities in their self-perception autonomy (@ 0.29) and resilience (@ 0.36) are reflected in: “Since I attend university, it will be easier for me to get a job” (1:45). This reinforces the position of Gillan and Coughlan (2010) who point out their ability to develop themselves when the opportunity is provided. 

In a negative sense, the main threat is manifested as hostility (@ 0.67) that emerges from their relationships with third parties (“Other people make fun of me” (1:43)), which should not be decisive for their development, since personal aspects or the disability itself should not affect social skills (Manzanera and Ortiz 2017), presenting an external limiting factor to their development (Sandoval et al. 2018) and the empowerment of their self-concept. 

This is added to the two main weaknesses that these subjects perceive, the different transversal skills (@ 0.27) to develop an ordinary life, and that they cannot develop due to their perception of their limitations (“I do not understand well people’s jokes ”(1:22)) and, in addition, the insecurity (@ 0.34) that they suffer when putting those skills into practice or in other common situations (“It is difficult for me to go shopping, I am not good with money” (1:117)). 

Therefore, the three factors that plenty of authors understand as intervening in the achievement of a positive self-concept (Brewer and Movahedazarhouligh 2019) are already present: the individual competence of the subject, the various opportunities that arise in the context for it (Manzanera and Brändle 2019), and the different props they may find (Arnaiz et al. 2020; Claiborne et al. 2010).

**Figure 4:**
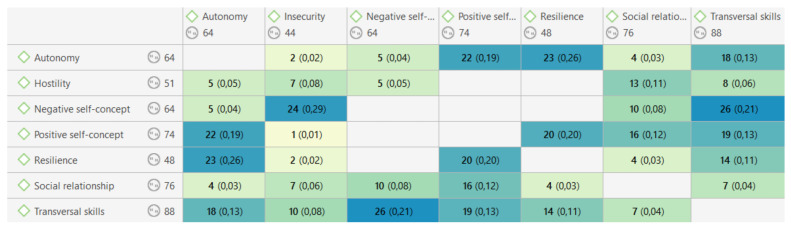
Analysis of relationships by code co-occurrence.

**Figure 5:**
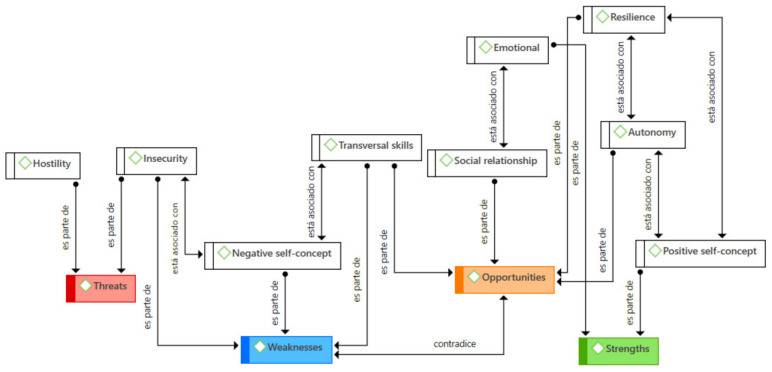
Semantic network with the main relationships between codes.

In a similar direction, it is also worth highlighting the different relationships established among the categories that emerged after the analysis (Figure 4), which led to different links (Figure 5), including taking part, associating, or contradicting, among others.

In the first place, the students’ self-concept is pointed out as the main codes that articulate the rest, since, apart from being some of those that appear with greater recurrence, they have a greater density of relationship with other codes. Specifically, when speaking of a negative self-concept, it is observed that it is directly related to transversal skills with a co-occurrence of @ 0.21, which are necessary for them in their day-to-day life and facilitate their inclusion and participation in society (Rocco 2011) (“I’m not good with technology, I don’t know how to handle e-mail nor some programs I don’t know how to use” (1:230)) and the insecurity (@ 0.29) that they present, in general, because of those skills that they must develop to perform basic tasks when attending university (Brewer and Movahedazarhouligh 2019), as someone said “I get overwhelmed when I don’t know how to do something like send e-mails”(1:229). 

On the other hand, there are some highlights when talking about their positive self-concept: their ability to be resilient (@ 0.20) (“I am capable of overcoming my fears” (1:79)) and the will they express to be autonomous and their capacity they have to do so (@ 0.19) (“I have been able to make my own decisions. I changed my professional profile” (1:52)), emphasizing the association established with that presence of resilience that characterizes them. A capacity to overcome adversity has already been reflected on other occasions (Moriña et al. 2020b) and that stands out in studies where subjects with and without disabilities are compared (Vehmas and Watson 2014). Besides, a contradiction arises between the different perceptions of themselves, when speaking of transversal skills, since they also express it as a potentiality (“I am good at mechanical work (Leroy Merlin)” (1:145)). Therefore, for their self-concept, the system of beliefs and attitudes in which they develop is shown as an added value, which, as previously indicated (Mullins and Preyde 2013), condition aspects such as their performance.

It is worth mentioning the appearance of two co-occurrences with a remarkable coefficient: (1) that of the codes assigned to quotes that refer to the subjects’ social relationships and the category established to refer to emotional aspects (@ 0.19) (“I am empathic, I put myself in the place of the people”(1:272), “I can be a good person with colleagues”(1:155)); (2) that of the quotes that were coded with categories referring to their autonomy and resilience (@ 0.26), with two characteristics that go hand in hand (“I will get a university certificate that will open doors for me” (1:252), “When I do my internships at the university, I will learn a trade” (1:110)). This is in line with the research that establishes an affective self-concept indicated by Claiborne et al. (2010), and that other authors (Ortiz and Olaz 2018; Fonseca et al. 2019) consider a fundamental element for their inclusion and where they feel safe when the environment is conducive to them (Desombre et al. 2019; Moriña et al. 2020a).

With the different relationships that were established at the conceptual level and observing the different emergent concurrences, the aforementioned semantic network was elaborated. This is described in Figure 5. It can be seen how the codes grouped in the upper left quadrant are related to the positive self-concept and the strengths and opportunities expressed, indicating the type of relationship that is manifested between each of them. On the contrary, in the opposite place are the codes that were used to indicate perceptions that refer to the weaknesses and threats that co-occur with that negative vision of their self-concept.

**Figure 6:**
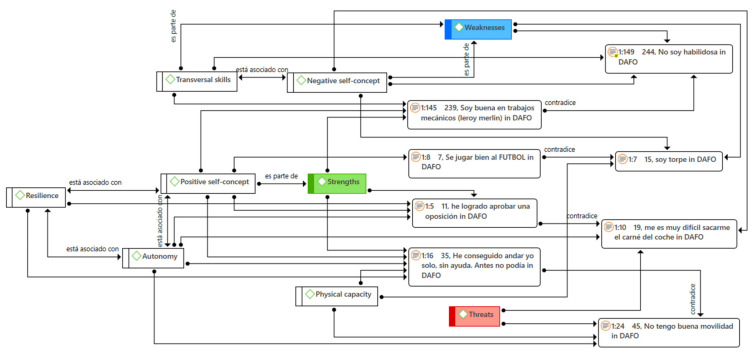
Semantic network of contradictions in SWOT perception.

As a last remark about the results presented here, a special mention should be made of the different contradictions that were found in the participants’ discourse and of which a sample is brought here (Figure 6). It may be an outline of another related aspect that would require a deepening of the investigation or, hypothetically, a way in which some of the emerged categories are manifested, such as the insecurity or the contradiction already indicated in the transversal skills through the different self-concepts, which could be the result of the hostile situations they suffer or the protectionism to which they are subjected.

(1) In the first of the cases, with quotes categorized as strengths and threats, it is noted that, on the one hand, autonomy is presented, emerging a positive self-concept (“I have managed to approve an opposition” (1:5)) and, on the other hand, how they lack this autonomy, negatively affecting their self-concept (“It is very difficult for me to get my car license” (1:10)). A possible explanation for this is the dependence, or not, of the result before external factors to the subject that may be negatively conditioning factors to the results obtained (Mock and Love 2012). Thus, although the fact of passing a public examination could initially be understood as more complex, it turns out to be a less difficult task for the subject, since passing it depends solely on the candidate who can control the rest of the variables (Fonseca et al. 2019). This does not happen this way when trying to obtain a driving license.

(2) With quotes that refer to the physical capacity of the subject, a second contradiction appears in the perception indicated as a strength and a weakness where the expression “I can play soccer well” (1:8) is incongruous with “I am clumsy” (1:7). That could be due to various factors, such as the same subject’s perception when carrying out one task and another (Ryan et al. 2019) since in soccer the fun factor arises. Thus, the negative vision is diminished (Ortiz and Olaz 2018). This is also the case when referring to his clumsiness, i.e., regarding the issues in which the aforementioned relaxed environment is not fostered.

(3) The third of the cases again refers to the physical capacity of the person, but other aspects that add complexity to the situation come into play, such as autonomy, resilience, and, in this case, strengths are expressed (“I managed to walk alone, without help. Before, I couldn’t” (1:16)). Threats are also expressed (“I don’t have good mobility” (1:24)). This shows how subjects can become aware of their limitations when performing one task or another (Sandoval et al. 2018), so it would be detrimental to their self-perception to prevent them from taking any action if they believe they are prepared for it (Langørgen and Magnus 2018; Moriña 2017), since it would not limit the autonomy they wish to acquire.

(4) There is another contradiction related to the transversal skills of the subjects. In this case, the subject presents a strength (“I am good at mechanical work” (1:145)) and a weakness (“I am not skilled” (1:149)), which are in opposition. This appreciation of their speech could be in line with what is reflected in the second point, where issues unconnected to them and external factors (Brewer and Movahedazarhouligh 2019) introduce variables that modify the opinion they have on the activities they develop (Grigal and Hart 2010; Mock and Love 2012).

## 4. Conclusions

This research has meant getting closer to the look that people with intellectual disabilities bring about themselves and how they perceive themselves based on different factors and problems that they must face in their day-to-day and in the near future. The most remarkable ones are those that refer to the different skills that they must possess to develop a life in a society that is as inclusive as possible and that facilitates their autonomy, as well as the importance of social relationships in self-concept. Regarding this, it is observed that the positive self-concept of the subjects dominated subtly. However, the two aspects of self-concept studied were repeatedly linked to the different weaknesses, threats, strengths, and opportunities that they expressed.

If the focus is placed on the objectives that were addressed, the following observations can be made.

- When exploring the perception of the factors that affect the self-concept of people with intellectual disabilities, it can be found that a total of eight categories have emerged. Among them, despite the substantial differences in their roots, it was observed that, to a greater or lesser extent, all of them are important for the development of the subjects and their inclusion in society. In addition, each one of them was present in a recurring way in the speech of all the subjects. Therefore, it can be concluded that, even though it is not a large sample, it has reached a theoretical saturation that allows the data to be extrapolated beyond this context.
- There was an objective to establish relationships between the categories emerged between the different factors that affect the self-concept of people with disabilities. Conceptually, a diversity of relationships developed between the codes used, both among those proposed before starting the data analysis process and among those that have emerged later. It can be inferred that there is a significant relationship between the different factors in which the subjects are involved and that an intervention from different angles, taking into account a diversity of aspects, is necessary.

In general, it can be asserted that the “We are all Campus” program contributes positively to the inclusion expectations of students, with a statement emphasizing this issue as recurrent on the part of the students. However, there is a need to carry out an evaluation that allows us to understand the effectiveness of this program and that these expectations are fulfilled and not just a wish that could generate frustration.

## Notes

1 As the work was originally carried out in Spanish, the codes used are in that language. Strengths—Fortalezas; Weaknesses—Debilidades; Opportunities—Oportunidades; Threats—Amenazas.

2 To reference the citations extracted from the data, the coding offered by the ATLAS.ti 9 program has been used. Where the first number indicates the document where the appointment is located and the second is the appointment within said document.

3 In the different figures facilitated by the ATLAS.ti software, the greater intensity of the relationships is reflected in proportionally darker colours.
